# SARS-CoV-2 genomic diversity and within-host evolution in individuals with persistent infection in the UK: an observational, longitudinal, population-based surveillance study

**DOI:** 10.1016/j.lanmic.2025.101154

**Published:** 2025-07-30

**Authors:** Mahan Ghafari, Steven A Kemp, Matthew Hall, Joseph Clarke, Luca Ferretti, Laura Thomson, Ruth Studley, The COVID-19 Infection Survey Group, The COVID-19 Genomics UK (COG-UK) Consortium, Ann Sarah Walker, Tanya Golubchik, Katrina Lythgoe

**Affiliations:** Pandemic Sciences Institute, University of Oxford, Oxford, UK; Big Data Institute, Nuffield Department of Medicine, University of Oxford, Oxford, UK; Big Data Institute, Nuffield Department of Medicine, University of Oxford, Oxford, UK; Pandemic Sciences Institute, University of Oxford, Oxford, UK; Big Data Institute, Nuffield Department of Medicine, University of Oxford, Oxford, UK; Office for National Statistics, Newport, UK; Nuffield Department of Medicine, University of Oxford, Oxford, UK; NIHR Health Protection Research Unit in Healthcare Associated Infections and Antimicrobial Resistance at the University of Oxford, Oxford, UK; NIHR Oxford Biomedical Research Centre, University of Oxford, Oxford, UK; Big Data Institute, Nuffield Department of Medicine, University of Oxford, Oxford, UK; The Sydney Infectious Diseases Institute (Sydney ID), School of Medical Sciences, University of Sydney, Sydney, NSW, Australia; Pandemic Sciences Institute, University of Oxford, Oxford, UK; Big Data Institute, Nuffield Department of Medicine, University of Oxford, Oxford, UK; Department of Biology, University of Oxford, Oxford, UK

## Abstract

**Background:**

Persistent SARS-CoV-2 infections in hospitalised immunocompromised individuals are known to facilitate accelerated within-host viral evolution, potentially contributing to the emergence of highly divergent variants. However, little is known about the evolutionary dynamics and transmission risks of persistent infections in the general population. We aimed to characterise the within-host evolution of SARS-CoV-2 during persistent infections identified through a large community surveillance study.

**Methods:**

We used data from the Office for National Statistics COVID-19 Infection Survey (ONS-CIS), a large-scale, longitudinal, population-based surveillance study conducted in the UK from April, 2020, to March, 2023. For this analysis, we focused on infections with high viral load (cycle threshold ≤30) and available genome sequences, from seven major SARS-CoV-2 lineages (alpha, delta, BA.1, BA.2, BA.4, BA.5, and XBB). ONS-CIS participants were randomly selected from the general population and tested regularly by RT-PCR, regardless of symptoms. We defined persistent infections as those with sustained or rebounding high viral RNA titres for 26 days or longer. We examined associated host characteristics and used raw sequence data to identify de novo mutations and estimate within-host synonymous and non-synonymous evolutionary rates across the SARS-CoV-2 genome.

**Findings:**

Between Nov 2, 2020, and March 21, 2023, we identified 576 persistent infections with at least two sequences, including 11 alpha, 106 delta, 102 BA.1, 204 BA.2, 16 BA.4, 133 BA.5, and 4 XBB. Persistent infections were more common in males than females (p<0·0001) and individuals older than 60 years (p=0·0027). The median within-host genome-wide evolutionary rate was 7·9× 10^−4^ substitutions per site per year (IQR 7·0–9·0× 10^–4^), with high interindividual variability driven largely by non-synonymous mutations, particularly in the N-terminal and receptor-binding domains of the spike protein. Longer infection duration was associated with higher evolutionary rates, while no associations were found with age, sex, vaccination status, previous infection, or virus lineage. We found no clear evidence of transmission beyond the first month of infection in any of the 84 persistent infections lasting 56 days or longer. In total, we identified 379 recurrent mutations, including many with known or predicted negative fitness effects and low prevalence at the population level, as well as de novo reversions to the Wuhan-Hu-1 reference sequence, which were likely under positive selection within those individuals.

**Interpretation:**

This study highlights the heterogeneous nature of within-host SARS-CoV-2 evolution in individuals with persistent infection in the community. Notably, a small subset of persistent infections with high viral loads underwent accelerated viral evolution or recurrently acquired hallmark mutations found in novel variants. In addition, onward transmission from a persistent infection during the later stages of infection is likely to be rare. These insights have important implications for prioritising genomic surveillance and managing patients with persistent infections.

**Funding:**

Department of Health and Social Care.

## Introduction

The evolutionary dynamics of SARS-CoV-2 has been marked by the emergence of highly divergent variants, including initial variants of concern alpha, beta, gamma, delta, and omicron, followed by second-generation omicron variants such as BA.2.75, XBB.1.5, and JN.1.^1–3^ A notable feature of these variants is that they have a large number of non-synonymous mutations compared with their closest ancestors, particularly in the spike protein’s N-terminal domain (NTD) and receptor-binding domain (RBD), and show signs of strong positive selection driven by increased transmissibility and antibody immune escape.^4,5^

Within-host evolution of SARS-CoV-2 likely plays a key role in shaping these patterns of evolutionary change over time. Many individuals with chronic SARS-CoV-2 infection also show evidence of strong viral adaptive evolution, characterised by accelerated evolutionary rates that feature key lineage-defining mutations in the spike protein.^1,6–8^ Given the probable importance of long-term (ie, persistent) infections on the evolution of the virus at the population scale, we sought to characterise the evolution of SARS-CoV-2 in persistent infections among the general population, identified as part of a multicentre community-based surveillance study, with sufficiently high viral loads for viral sequences to be obtained.

The majority of studies on the evolutionary dynamics of persistent SARS-CoV-2 infections have focused on persistent-chronic cases. These are infections with consistently high viral titres (cycle threshold [Ct] ≤30) and positive PCR results and are often found in hospitalised patients who are immunocompromised and receiving treatments. This focus on persistent-chronic infections is partly because infections that may occur at very low viral load levels are challenging to identify and manage clinically.^9^ However, unlike earlier studies conducted between 2020 and early 2024, which primarily focused on hospitalised patients, we recently showed that persistent SARS-CoV-2 infections—many of which have rebounding viral loads—are also prevalent inthe generalpopulation.^10^ There remainsa major gap in our understanding of host factors contributing to higher odds of experiencing persistent SARS-CoV-2 infection, reasons why the virus undergoes accelerated adaptive evolution in certain individuals, but not in others, identifying genomic regions and mutations, particularly outside of the spike gene, that undergo adaptive evolution during persistent infections, and ultimately developing effective therapeutics to clear viral infections.^11,12^ Characterisation of evolution is particularly important to determine whether adaptive changes during infections mirror the saltatory evolution of SARS-CoV-2 observed with the emergence of new, highly divergent variants. Additionally, identifying mutations that present complex trade-offs, being advantageous at the within-host level but detrimental at the between-host level, is crucial for understanding evolutionary factors that contribute to prolonged viral replication within hosts and increased odds of transmission between hosts.^6,13^

Here, we explored the within-host evolutionary dynamics of SARS-CoV-2 in individuals with persistent infection and identified factors associated with rate differences between individuals. Investigating the evolutionary dynamics of SARS-CoV-2 within persistent infections is essential for understanding the selective pressures that shape viral evolution at the within-host level and factors contributing to increased risk of resistance to treatments, and also to gauge the extent to which these infections might lead to onward transmission of the virus to the rest of the population and contribute to the generation and subsequent spread of new variants.^14–16^

## Methods

### Study design and participants

We used data from the Office for National Statistics COVID-19 Infection Survey (ONS-CIS), which was an observational large-scale, population-based, household-based study in the UK designed to monitor SARS-CoV-2 infection and immunity trends in the community. The survey was launched in England on April 26, 2020, and was expanded to include Wales on June 29, 2020, Northern Ireland on July 26, 2020, and Scotland on Sept 21, 2020,^17^ and closed on March 31, 2023.^2^ Households from nationwide address lists were randomly selected and invited to participate by letter, ensuring as representative a cross-section of the population as possible. All household members aged 2 years and older were eligible for inclusion in ONS-CIS. Written informed consent was obtained from all adult participants; for those younger than 16 years, consent was provided by a parent or legal guardian. Participants gave written informed consent to contribute swab samples (self-collected or by a parent or carer for those younger than 12 years), irrespective of symptoms, and completed a questionnaire for each assessment. No race or ethnicity data were collected as part of this study. All versions of the study protocol and questionnaires are available online.

For the study protocol see https://www.ndm.ox.ac.uk/covid-19/covid-19-infectionsurvey/protocol-andinformation-sheets

Approximately 98% of the participants in the survey consented to routine PCR sampling at weekly intervals for the first month of enrolment and monthly thereafter for the duration of the study.^2,10^ The survey offered participants the option of only having one enrolment assessment (taken by approximately 1%), or weekly assessments for only 1 month (taken by approximately 1%). From the start of the survey to Nov 31, 2020, sequencing was attempted for a random subsample where a participant tested positive with a high viral load (Ct≤30), with additional retrospective sequencing of stored samples, resulting in approximately 20–40% of positive samples being sequenced each week—the exact proportion of sequenced samples during this period varied over time depending on laboratory capacity.^2^ The subsample was selected centrally by the COVID-19 Genomics UK (COG-UK) Consortium without reference to the survey (ie, from all positive PCR tests). From Dec 1, 2020, onwards, in response to the emergence of the alpha variant, the decision was made to attempt sequencing of all RT-PCR-positive samples from the survey with a Ct value of 30 or below through the COG-UK Consortium. The cutoff for Ct value was chosen because at higher Ct values (corresponding to lower viral loads) sequencing success was less likely.

Ethical approval for the study was granted by the South--Central Berkshire B Research Ethics Committee (reference 20/SC/0195).

For the questionnaires see https://www.ndm.ox.ac.uk/covid-19/covid-19-infectionsurvey/case-record-forms

### Sample collection

From April 26, 2020, to July 31, 2022, assessments were conducted by study workers visiting each household; from July 14, 2022, onwards, assessments were remote, with swabs taken using kits posted to participants and returned by post or courier, and questionnaires completed online or by telephone. During the remote phase, participants self--administered nose and throat swabs during scheduled visits, with swabs for children younger than 12 years collected by a parent or carer. Samples with high viral loads (Ct≤30) were sent for whole-genome sequencing at one of the networks of laboratories contributing to the COG-UK Consortium.

### Identification of persistent infections

We defined persistent SARS-CoV-2 infections as infections with at least two RT-PCR-positive samples with a high viral RNA titre (Ct≤30), collected at time intervals of at least 26 days apart, and representing the same infection. We used the consensus sequences generated using ARTIC Nextflow (version 1) or Shiver (version 1.5.8) (appendix 1 p 2) to determine whether two or more sequences from the same individual were from the same infection, using the method outlined in our previous work.^10^ Briefly, if two sequences from the same individual were collected at least 26 days apart, were of the same major lineage, and shared a rare single nucleotide polymorphism (SNP) compared with the population-level consensus, the individual was determined to have persistent SARS-CoV-2 infection. Our analysis coveredinfectionswith the alpha, delta, BA.1, BA.2, BA.4, BA.5, and XBB major lineages, which were preselected as they represented the most prevalent SARS-CoV-2 lineages in the UK and were associated with distinct waves of infection during the study period. An SNP was deemed to be rare if found in fewer than 400 samples of that lineage (appendix 1 p 13). Due to possible misclassification of some BA.2 sequences as BA.5 and vice versa using the Pango lineage nomenclature,^18^ we considered the possibility that some BA.5 sequences could belong to a BA.2 infection.

### Identifying intra-host single nucleotide variants

We called an intra-host single nucleotide variant (iSNV) at a given position in the genome if there were ten or more bases called at that position, including gaps, and if the most common minor allele was present at 20% or more but less than 50% of the total bases at that position. The small number of bases required to call an iSNV was chosen because many samples had low viral titre, while the 20% threshold was to avoid biases introduced by differing amounts of sequencing noise across all the samples.

We also identified mutations, which we defined as iSNVs or major alleles that differed from the majority allele at the first sampling timepoint, and reached at least 20% frequency at the first timepoint (hereafter referred to as baseline) or any of the subsequent timepoints. Whereas iSNVs are always less than 50% frequency by definition, a mutation can be at any frequency above 20% (including 100%). To ensure consistency of methods across our analyses, we also defined the majority-rule consensus at each sampling as the majority allele, with a minimum of ten bases to call a consensus at any given position. Unless stated otherwise, when we refer to the consensus we mean the majority-rule consensus, not the consensus generated using ARTIC Nextflow or Shiver. Mutations in the coding region are classed as being non-synonymous if they induce a codon change relative to the baseline consensus, using the SARS-CoV-2 Wuhan-Hu-1 genome (accession number: NC_045512.2) as the reference sequence to define the genomic positions of the coding genes.

Some positions in the genome are prone to having low-frequency iSNVs in a high proportion of samples and are often sequencing centre-specific. Although we do not know what causes these low-frequency iSNVs, they are unlikely maintained through descent and we therefore label them artefactual iSNVs. For each sequencing centre in our study, we masked genomic positions where an iSNV was present at 2% or higher frequency in more than 1% of samples from that sequencing centre. The iSNVs did not include the primer-binding regions.

### Data analysis

We calculated within-host nucleotide diversity of the virus using the π statistic (appendix 1 p 3). We measured differences in mutant allele frequencies between two sequences from the same infection to estimate the genetic distance between the sequences (appendix 1 pp 3–4) and then used linear regression models to measure virus evolutionary rates and stochastic changes in allele frequencies due to sequencing noise (appendix 1 pp 4–6). For this analysis, we excluded sample pairs where the total number of overlapping base pairs between the two consensus sequences was less than 50% of the genome length. This criterion was applied to prevent inflated or deflated measures of genetic distance per site. Additionally, we excluded any low-coverage sequences with less than half the genome length covered. To identify the most suitable model for measuring within-host evolutionary rates, we compared several linear regression models of varying complexity using their Bayesian information criterion (BIC) values. In these models, the slope of the regression line represents the rate of evolution, while the y-intercept indicates the level of background noise in the data (appendix 1 pp 5–7). To measure the between-host evolutionary rate of different major lineages (including the within-lineage and between-lineage rates), we first constructed the ancestral sequence for each major lineage and then calculated the Hamming distances between samples from each major lineage relative to the ancestral sequence of the same major lineage (appendix 1 pp 6–7). To calculate the divergence rate across the virus genome, we first assumed that the majority-rule consensus sequence at the first timepoint of each persistent infection is the founding virus and estimated the start time of infection as the midpoint between the last negative PCR test and the first sequence from the persistent infection (appendix 1 p 14). This allowed us to measure divergence from the putative founder across all individuals for each segment of the genome (appendix 1 pp 7–8).

### Role of the funding source

The funder of the study had no role in study design, data collection, data analysis, data interpretation, or writing of the report.

## Results

**Figure 1:**
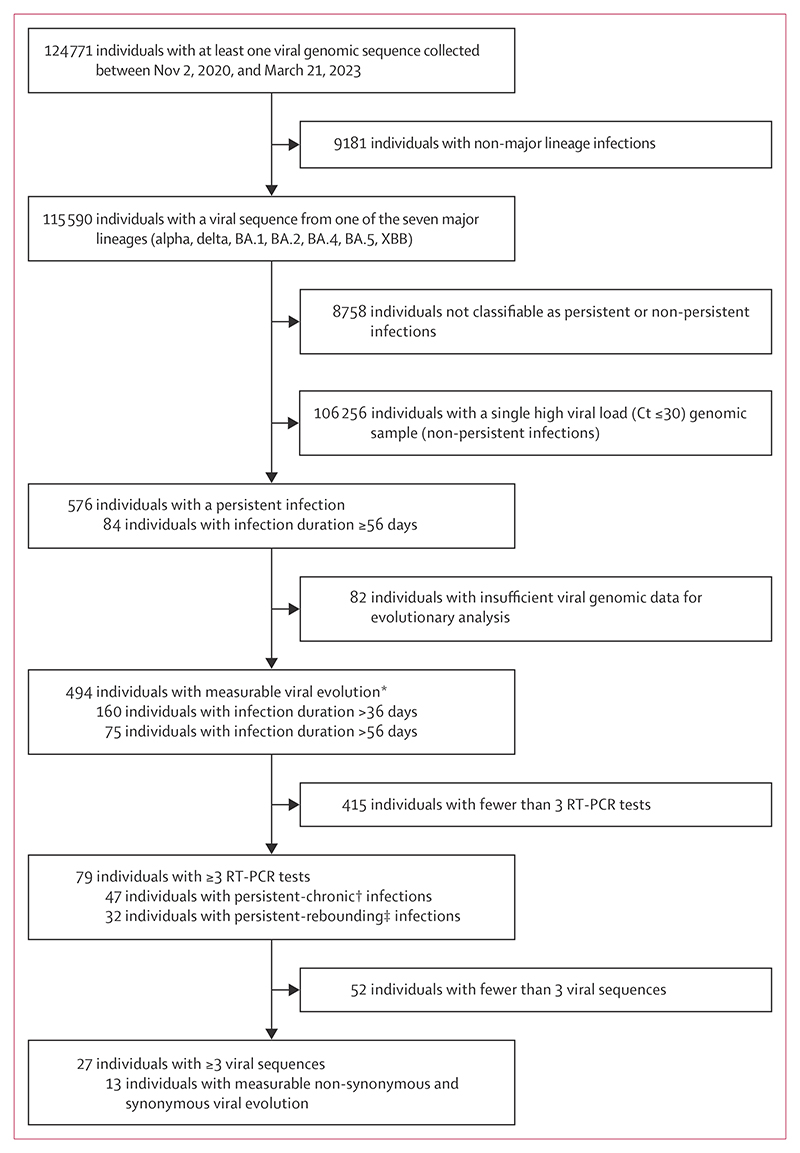
Flowchart of COVID-19 Infection Survey participants in this study Ct=cycle threshold. *This refers to any persistent SARS-CoV-2 infection with sufficient data to report an evolutionary rate, specifically those with at least one intra-host single nucleotide variant present at ≥ 20% frequency at any timepoint. Sample pairs with <50% genome overlap were excluded. †SARS-CoV-2 infections with consistently positive RT-PCR test results. ‡SARS-CoV-2 infections with at least one negative RT-PCR test result.

We limited our analysis to samples collected between Nov 2, 2020, and March 21, 2023, corresponding to the period following the emergence and early spread of the alpha variant in the UK in late 2020. We identified 115 590 infections from 83 981 households in the ONS-CIS who had at least one RT-PCR-positive sample with high viral load (Ct≤30) and a corresponding viral genome sequence belonging to one of seven preselected major SARS-CoV-2 lineages (alpha, delta, BA.1, BA.2, BA.4, BA.5, or XBB). From this group, we identified 576 persistent infections, defined as those with high viral load viral sequences sustained or rebounding for 26 days or longer and confirmed using the sequence data (figure 1).

We identified three cases of BA.2 persistent infections, which included at least one sequence misclassified as a BA.5 lineage (see Methods). Without requiring any additional adjustment to separate second-generation BA.2 (eg, BA.2.75) and BA.5 (eg, BQ.1) major lineages from their closest ancestors, our method reliably recovered subsets of infections within BA.2 and BA.5 that were attributable to second-generation variants.

The median duration of these persistent infections—measured as the number of days between the first and last sequenced PCR-positive samples—was 31 days (IQR 28–39). 84 infections lasted 56 days or longer (figure 1). These durations reflect only timepoints with sequenced samples and likely underestimate the full PCR-positive period. Baseline characteristics of participants, including age, sex, and viral lineage, are summarised in the table.

**Figure 2:**
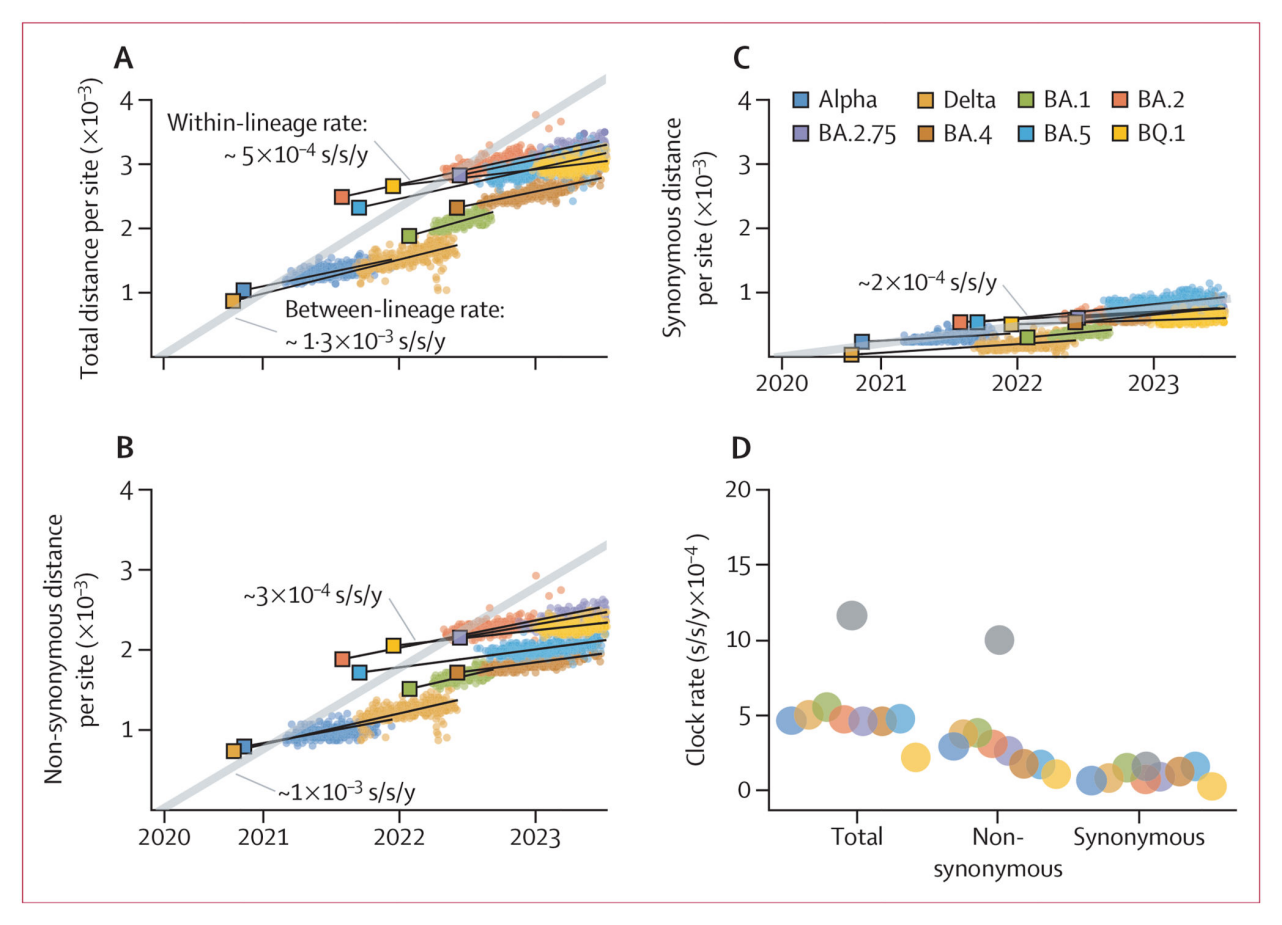
Evolutionary dynamics of SARS-CoV-2 at the between-host level (A) Mutations accumulate linearly over time within each major viral lineage, punctuated by significant evolutionary leaps that demarcate these lineages (between-lineage rate; grey line). This pattern is characterised by a disproportionate accumulation of non-synonymous mutations at the point of transition between major lineages (B), whereas synonymous mutations accumulate at a comparatively steady rate both within and across these lineages (C). Genetic distance within each major lineage is the Hamming distance between the putative ancestral sequence (shown with square markers) of that major lineage. The between-lineage distance is calculated as the Hamming distance between Wuhan-Hu-1 reference sequence (NC_045512.2) and the putative ancestors of each major lineage. Lines represent the best fit from a linear regression. (D) Substitution rate per site per year for genome-wide (total), non-synonymous, and synonymous mutations, over time per major lineage. The substitution rates are 2·5–6·0× 10^−4^ s/s/y for genome-wide, 1·5–4·0× 10^−4^ s/s/y for non-synonymous, and 0·5–2·5× 10^−4^ s/s/y for synonymous mutations per major lineage. The between-lineage rate is highlighted with grey circles. The source data for this analysis are derived from a previously identified representative set of consensus sequences from the Office for National Statistics COVID-19 Infection Survey dataset.^2^ s/s/y=substitutions per site per year.

Our analysis of the evolutionary dynamics of SARS-CoV-2 at the between-host level identifies two distinct patterns of mutation accumulation: within-lineage and betweenlineage rates. Within each major viral lineage, mutations accumulate linearly over time, indicating a steady evolutionary clock (figure 2). The within-lineage rate is characterised by non-synonymous and synonymous mutations accruing at relatively similar rates. Taking synonymous mutations as a proxy for neutral changes, this suggests that the within-lineage evolution is neutral or nearly neutral. However, the evolutionary pattern is punctuated by significant leaps at the points of transition between major lineages^19,20^ (figure 2). Similar to the findings from a previous study,^20^ we found that these transitions show a much higher rate of accumulation of non-synonymous mutations compared with synonymous ones (grey line in figure 2A–C), indicating bursts of adaptive evolution that distinguishes one major lineage from another.

**Figure 3:**
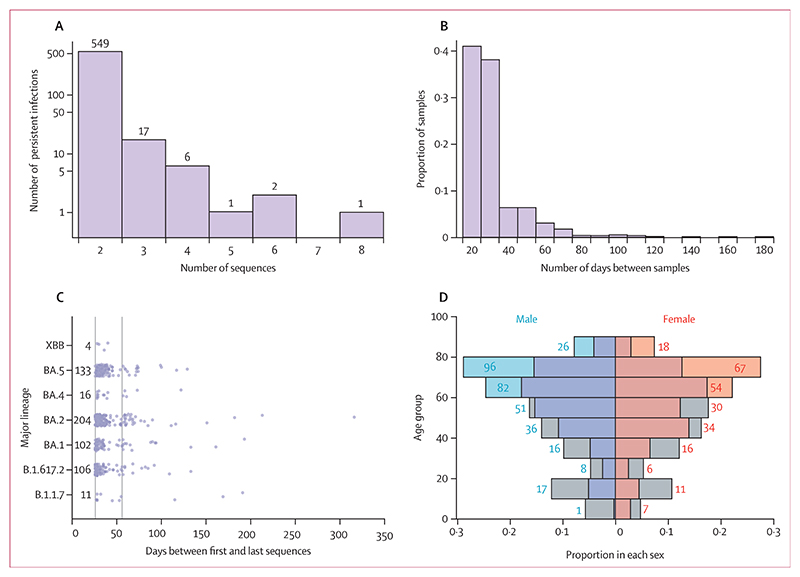
Distribution of age, sex, infection duration, number of sequences, and lineage among individuals with persistent SARS-CoV-2 infections (A) Number of sequences per persistent SARS-CoV-2 infection. Numbers on each bar show the number of persistent infections per category. (B) Distribution of numbers of elapsed days between consecutive sequences collected per persistent SARS-CoV-2 infection. In people with persistent SARS-CoV-2 infection who gave multiple samples, each pair of consecutive samples is considered. (C) Number of days between the earliest and latest genomic samples for each persistent infection, with each point representing a persistent infection. Solid vertical lines are drawn at the 26-day and 56-day marks to denote the thresholds for persistent infections lasting at least 1 month and 2 months, respectively. Numbers on the side of each bar show the total number of persistent infections per major lineage. (D) Proportion of persistent infections in each sex and per age group. Numbers on each bar show the raw number of persistent infections in each age group. Grey bars on either side show the relative proportion of infections with a single positive PCR within the Office for National Statistics COVID-19 Infection Survey per sex and age group.

To better understand how the host characteristics and evolutionary dynamics of SARS-CoV-2 at the between-host level relate to those within hosts, we further investigated the virus’s evolutionary dynamics during persistent infections. All 576 persistent infections had viral sequencing data from at least two timepoints; 27 had sequencing data from three or more timepoints, typically collected at 20–40-day intervals, and the longest-lasting persistent infection spanned nearly a year with eight sequenced timepoints (figure 3A–C). After reviewing the PCR test histories and conducting a phylogenetic analysis of consensus sequences from all household members of persistent infection cases, none of the 84 persistent infections lasting 56 days or longer showed clear evidence of transmission beyond the first month of infection. This suggests that late transmission events from persistent infections, if they occur, are likely very rare.

Compared with individuals with a single high viral load positive PCR and an associated viral sequence within the ONS-CIS (hereafter referred to as non-persistent infections), individuals with persistent infection were more prevalent in the age groups above 60 years (*χ*^2^=8·98, df=1, p=0·0027; figure 3D). We also found a significant association between sex and type (ie, persistent *vs* non-persistent) of infection (*χ*^2^=21·28, df=1, p<0·0001), with males representing 58% (333 of 576) of people with persistent SARS-CoV-2 compared with 48·1% (51 130 of 106 256) of people with non-persistent SARS-CoV-2. There was no strong evidence of association between infection type and SARS-CoV-2 lineages (*χ*^2^=9·9218, df=5, p=0·077; also see appendix 1 p 2).

We then investigated the within-host evolutionary dynamics of the virus in these 576 individuals by first identifying iSNVs for each sample collected during infection, and measuring nucleotide diversity, π, over time.

**Figure 4:**
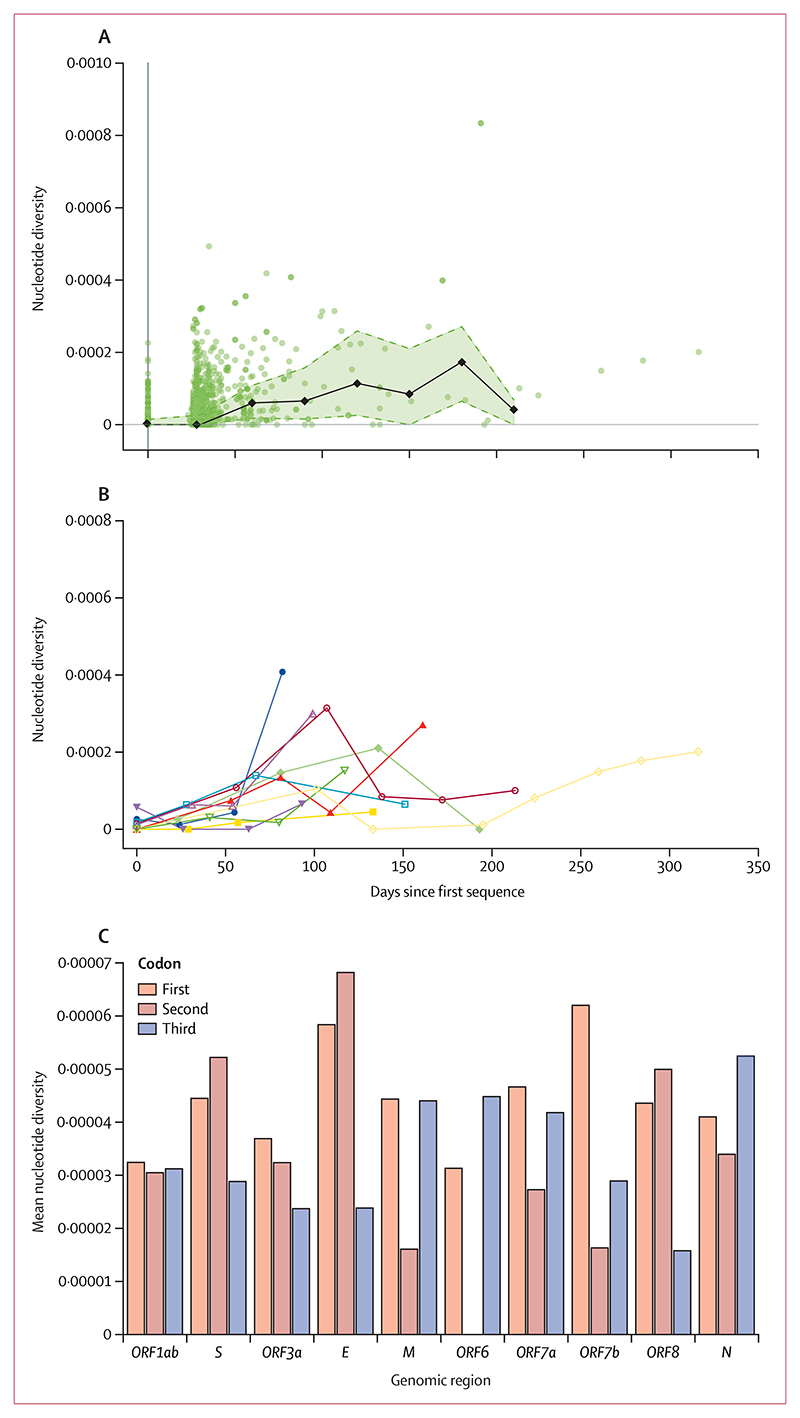
Within-host nucleotide diversity (A) Aggregate nucleotide diversity (π) over time across all persistent infections. Each datapoint represents the diversity of a sample from a persistent infection at a given time since the first sequenced sample in that infection (t*=*0). The black line shows the median nucleotide diversity in 30-day intervals and the shaded area covers the IQR. (B) Nucleotide diversity over time for persistent infections with three or more samples. (C) Mean nucleotide diversity per codon position in each genomic region including the open reading frames (ORFs), spike (*S*), envelope (*E*), membrane (*M*), and nucleocapsid (*N*).

In the majority of cases, nucleotide diversity at the earlier timepoints for each persistent infection was very low, with 355 (62%) of 576 infections displaying no detectable diversity at baseline, and a gradual increase in diversity at later timepoints (figure 4A). This suggests that the first sample in most persistent infections was collected near the onset of infection, and with infection initiated by a single, or very closely related variants.^21,22^ We did not find any strong evidence for co-infection, super-infection, and within-host recombination during any of the persistent infections. This can, in part, be explained by the low genetic diversity observed in our samples after quality control (masking artefactual sites and applying a 20% frequency cutoff for iSNVs).

There was wide variation in diversity over time across different infections (figure 4B), with the average within-host diversity of all sampling timepoints being approximately 4 × 10^−5^ per nucleotide, which is more than an order of magnitude smaller than the between-host diversity at approximately 5 × 10^−4^ per nucleotide.^2^

We also measured nucleotide diversity by codon position. The first and second codon positions typically induce non-synonymous changes, while most iSNVs in the third position result in synonymous changes.^23^ Looking at the first and second position across different genomic regions within our samples, the lowest nucleotide diversity was in *ORF6*, with no diversity at the second position, indicating this genomic region is highly conserved and likely subject to strong purifying selection. Conversely, the envelope (*E*) gene showed the highest diversity at the first two codon positions, followed by spike (*S*) and *ORF8* (figure 4C). Some of the other genomic regions such as *ORF1ab* had a more uniform diversity across all three codon positions while *ORF6* and nucleocapsid (*N*) had higher synonymous diversity compared with non-synonymous diversity across all genomic regions.

**Figure 5:**
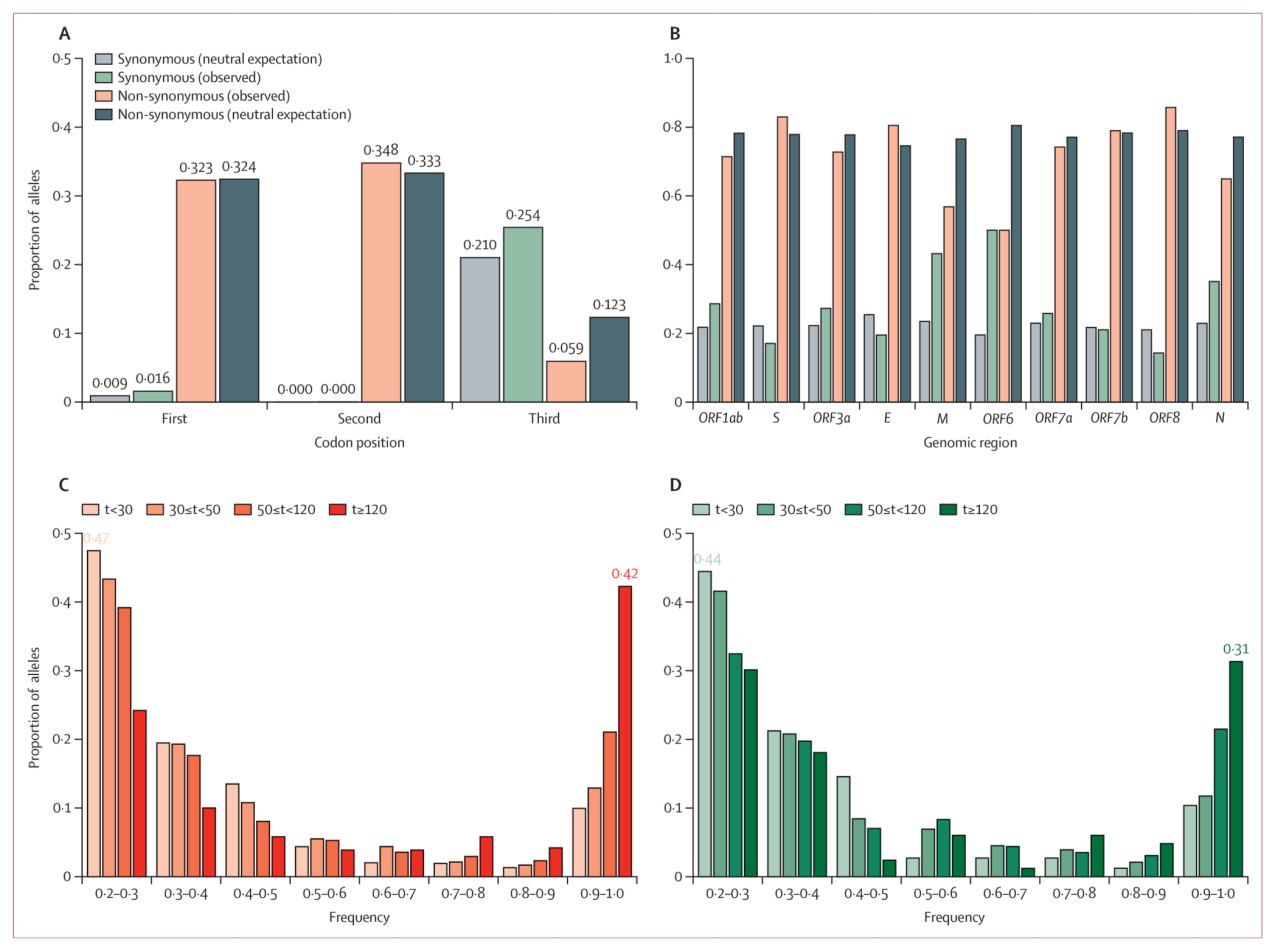
Basic characteristics of mutant alleles (A) Proportion of synonymous (green) and non-synonymous (orange) mutant alleles per codon position observed in samples from persistent infections, taking the majority allele at the first timepoint as reference, compared with expectations under neutrality, taking NC_045512.2 as reference. (B) Proportion of alleles per mutation type for each genomic region including the open reading frames (ORFs), spike (*S*), envelope (*E*), membrane (*M*), and nucleocapsid (*N*). (C–D) Proportion of synonymous (C) and non-synonymous (D) alleles over time across different frequency bands. The proportions of alleles within the smallest and largest frequency bands are highlighted for both early (t<30 days) and late (t≥120 days) stages of SARS-CoV-2 infection. t=time since first SARS-CoV-2 genomic sample per infection.

Next, we identified synonymous and non-synonymous mutations present at 20% frequency or above at any timepoint over the course of each infection, taking the majority allele at the first timepoint as reference. 2551 (73%) of all 3486 mutant alleles within the coding region were non-synonymous, with 57 (2%) synonymous at the first and second codon positions (figure 5A). *ORF6*, membrane (*M*), and *N* had the highest proportion of synonymous compared with non-synonymous mutations, and *ORF8* the lowest (figure 5B). Comparing the allele frequency of mutations at different points during infections, towards the start of infections (<120 days since baseline), both non-synonymous and synonymous alleles were typically at comparable frequencies (figure 5C, D). However, later on (>50 days since baseline), a higher proportion of non-synonymous alleles appeared at higher frequencies—42% of non-synonymous alleles were present above 90% frequency compared with 31% of synonymous alleles—indicative of positive selection (figure 5C, D).

Non-synonymous alleles were 2–3 times more prevalent than synonymous alleles across all frequency bands (appendix 1 p 15), with 2545 (73%) of 3486 mutants in the coding region that exceeded 50% frequency being non-synonymous. This ratio is close to the expectation under neutrality, with 78% of all possible mutations across the genome expected to be non-synonymous^23^ (figure 4A, B). Given it has previously been found that half of the mutations causing non-synonymous changes are purged both at the between-host level and during acute infections (dN/dS ≈ 0·5),^21,24^ observing a ratio of non-synonymous to synonymous mutations that is similar to the neutral expectation in individuals with persistent SARS-CoV-2 infection suggests that at least some genomic regions are under positive selection.

To determine the within-host evolutionary rates for each infection, we used changes in allele frequency relative to baseline as a proxy for measuring evolutionary distance over time. We excluded 82 (14%) of 576 persistent infections from the evolutionary rate analysis because the paired consensus sequences from baseline and asubsequent timepoint in these cases shared less than 50% of the genome in overlapping base calls. The remaining 494 infections were classified as those with measurable evolution (appendix 1 p 16).

**Figure 6:**
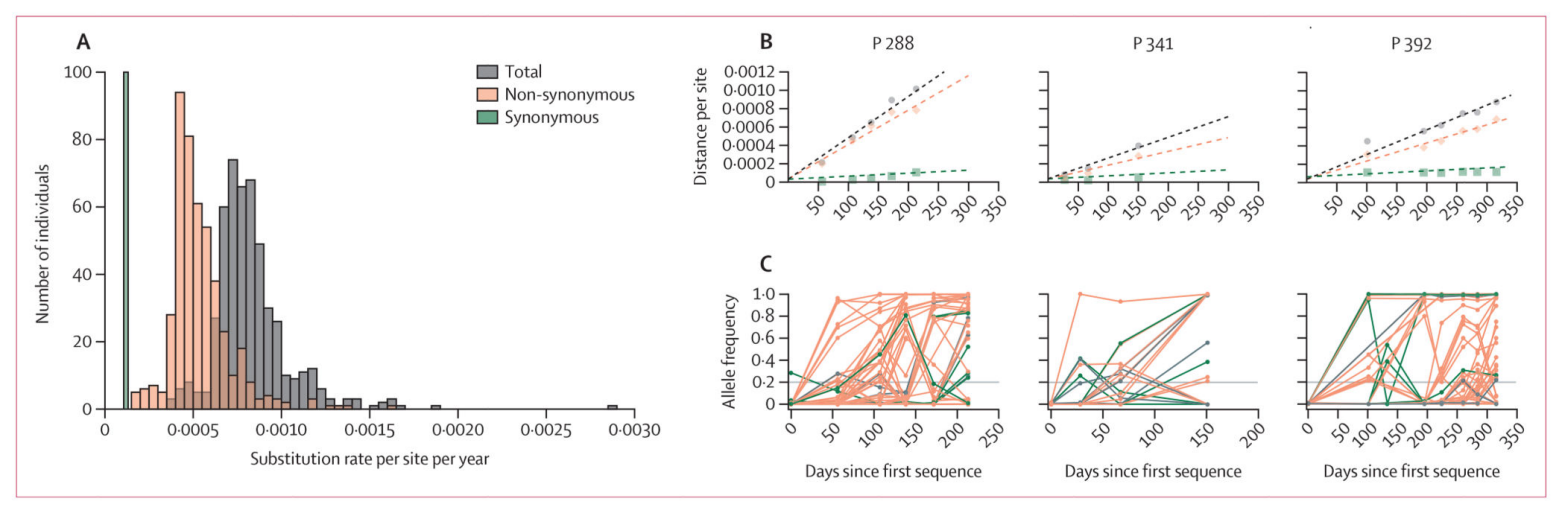
Rates of genome-wide, non-synonymous, and synonymous evolution in individuals with persistent SARS-CoV-2 infection (A) Distribution of inferred evolutionary rates per individual for the 494 persistent infections with measurable evolution, based on analyses using a linear mixed-effects model optimised for the best fit to the data (as indicated by the lowest Bayesian information criterion value). The model differentiates between unique genome-wide (grey) and non-synonymous (orange) rates for each individual, while applying a single synonymous rate (green) across all individuals. (B) Evolutionary distance over time for three selected individuals with persistent SARS-CoV-2 infections—see the appendix 1 (pp 20–29) for all persistent infections. Points on the graphs represent the total genetic distance from the consensus sequence at the initial timepoint, calculated based on allele frequency changes over time. Points are not shown (and genetic distances not calculated) for timepoints where the sequence from that timepoint shares less than 50% consensus genome overlap with the sequence from the first timepoint (day 0). Dashed lines indicate the regression lines that best fit these data. (C) Mutant allele frequency trajectories for the three persistent infections examined, categorised into synonymous, non-synonymous, and non-coding (grey) mutations—see appendix 1 p 17 for trajectories in all individuals with measurable evolution with at least three timepoints. Each mutation that reached a minimum frequency of 20% at least at one timepoint is shown. We can see partial and full sweeps of de novo mutations over the course of persistent infections. A horizontal grey line across the graphs marks the 20% allele frequency threshold.

We found significant variation in genome-wide and non-synonymous evolutionary rates among individuals, whereas synonymous rates remained largely consistent (appendix 1 p 9). We also confirmed that the noise in allele frequencies was not associated with differences between sequencing centres (appendix 1 p 9). The median genome-wide evolutionary rate was 7·9× 10^−4^ substitutions per site per year (s/s/y) with an IQR of 7·0–9·0× 10^−4^ s/s/y (figure 6A). 469 (95%) of 494 persistent infections showed an evolutionary rate exceeding 5·5× 10^−4^ s/s/y, indicating that the vast majority of individuals experienced a rate surpassing the between-host within-lineage evolutionary rate of SARS-CoV-2, which typically ranges from 2·5× 10^−4^ to 5·0× 10^−4^ s/s/y for the alpha, delta, and omicron sublineages (figure 2). Furthermore, 11 (2%) of the 494 infections had an evolutionary rate higher than the between-lineage rate of 1 × 10^−3^ s/s/y. The rate of non-synonymous evolution was 5·0× 10^−4^ s/s/y (IQR 4·4–6·1× 10^−4^), which was about four times higher than the synonymous rate of 1·2× 10^−4^ s/s/y across more than half of the persistent infections (figure 6).

The considerably higher rate of non-synonymous evolution indicatesthat at least some non-synonymousmutations are subject to positive selection, and moreover that this selective pressure differs among individuals. In contrast, the preference for a regression model with a single rate for synonymous mutations implies that these mutations are evolutionarily neutral or nearly neutral, evolving at approximately the same rate across all individuals. Our within-host synonymous rate estimate is consistent with the between-host synonymous rate (figure 2). After adjusting for the proportion of sites available for synonymous mutations (approximately 22%), the synonymous rate becomes 1·5× 10^−6^ substitutions per site per day, broadly consistent with mutation rate estimates for SARS-CoV-2 and other betacoronaviruses.^1^

To confirm the quality of fit of our regression model, we visualised patterns of divergence for the 13 persistent infections that had a strong clock-like evolutionary signal, at least one synonymous mutation, and at least one non-synonymous mutation (figure 1; appendix 1 p 16), Among these infections, non-synonymous mutations more frequently reached high frequencies compared with synonymous ones (appendix 1 p 16; see also figure 6B, C). We also observed patterns of transient alleles emerging and disappearing across many of these 13 infections, possibly indicating the presence of distinct subpopulations within infections (appendix 1 p 17).

We found no significant associations (ΔBIC <0) of age, sex, vaccination status, previous infection, virus lineage, and experiencing persistent-chronic versus persistent-rebounding infectionswith within-host viral evolution rates. This evaluation was based on comparing the BIC values of the best-fit regression model for determining within-host rates with models that included each of these parameters as an additional fixed effect (appendix 1 p 10). However, we did identify a positive association (ΔBIC >2) between the evolutionary rates and the duration of infection, indicating that longer infections show higher rates of non-synonymous evolution. To determine if this association was biased by the lower genetic diversity typically seen in shorter infections, which could result in lower evolutionary rate estimates, we also examined the 160 infections lasting longer than 36 days and 75 infections lasting longer than 56 days with measurable evolution (figure 1). Our analysis confirmed statistical support (ΔBIC >2) for the positive relationship between infection duration and evolutionary rates, even within these subsets of infections (appendix 1 p 10).

**Figure 7:**
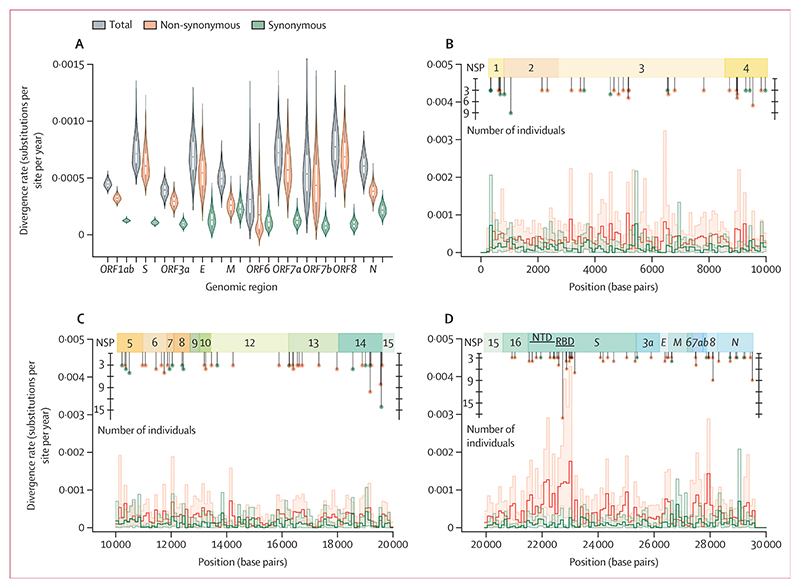
Virus divergence rates across the genome (A) Estimated divergence rates from the putative founder, including genome-wide (grey), non-synonymous (orange), and synonymous (green) substitution rates across different genomic regions including the open reading frames (ORFs), spike (*S*), envelope (*E*), membrane (*M*), and nucleocapsid (*N*). The distributions represent the bootstrap estimates derived from 576 persistent infections. (B–D) Estimated divergence rate per 100 (non-overlapping) base pair segments of the genome for NSPs: NSP 1 to 4 (B), NSP 5 to 15 (C), and NSP 15 and 16, along with other structural and accessory proteins (D). Shaded area represents the 95% CIs from bootstrapping. Recurrent mutations identified in three or more persistent infections are highlighted. NSP=non-structural protein. NTD=N-terminal domain. RBD=receptor-binding domain.

To explore patterns of evolution among different regions of the genome we aggregated the data for all 494 persistent infections with measurable evolution since there was insufficient signal to measure evolution by genomic region per individual infection. We observed considerable variability in divergence rates across the genome (figure 7). The bulk of this rate variation came from non-synonymous changes, with the rate of synonymous divergence remaining relatively uniform across most regions, except for the *M* and *N* genes which had a synonymous rate nearly double that of the other regions (figure 7A). *ORF8* and *S* had the highest rates of non-synonymous divergence, nearly five times greater than the rates of synonymous divergence, whereas *ORF6* showed the lowest rate of non-synonymous divergence, further indicating that it is likely under strong purifying selection.

To investigate divergence rates at a finer scale than gene, we also considered non-overlapping gene segments of 100 base pairs in length across the whole genome. Most segments in *ORF1ab* and *S*, which together make up approximately 85% of the SARS-CoV-2 genome, displayed low levels of variation in synonymous divergence rates, while non-synonymous rates varied up to five times in some segments of *ORF1ab*, and ten times in *S* (figure 7B–D). The end tail of the RBD in *S* (nucleotide positions 22 990 to 23 090) had the highest rates of non-synonymous divergence, suggesting that it is under the strongest positive selection (figure 7D).

We found 379 (262 non-synonymous and 117 synonymous) mutations in at least two individuals among the 576 persistent infections (appendix 2; see also figure 7B–D). The highest concentration of these recurrent mutations—calculated by dividing the number of mutations by the length of the genomic region—that were non-synonymous were in *ORF8* (24 mutations), *E* (14 mutations), and *S* (210 mutations), whereas the highest concentration of recurrent synonymous mutations was in *ORF7b* (three mutations) and *M* (14 mutations). Considering alternative reading frames, we found that *ORF9b*, embedded within the *N* reading frame, has a notable impact on the interpretation of mutations. Specifically, 11 synonymous mutations within the *N* reading frame are non-synonymous with respect to *ORF9b*, which partially explains the elevated synonymous divergence observed for *N*. In contrast, *ORF3c*, overlapping with *ORF3a*, had a much smaller effect, converting only four synonymous mutations in *ORF3a* to non-synonymous mutations in *ORF3c*.

We were next interested inthe between-host fitness effects of the mutations found in the persistent infections, by using previously published estimates of the between-host perlineage fitness effects of mutations, which used a globally representative SARS-CoV-2 phylogeny.^25^ When controlling for lineage, of the 2938 mutations we observed in total, 870 (30%) had a fitness advantage at the between-host level, which fell to 682 (27%) of 2559 for only non-recurrent mutations (appendix 1 p 18). However, for the 379 recurrent mutations that emerged in multiple individuals, 188 (50%) had a positive fitness effect at the between-host level (appendix 1 p 18). Notably, many recurrent mutations also had very low population prevalence in the major lineage in which they appeared at least once during persistent infection. Specifically, 310 (47%) of 658 recurrent mutation—lineage combinations were present in less than 0·01% of all ONS-CIS samples from the same major lineage (appendix 1 p 18). This finding suggests that almost half of the recurrent mutations have a fitness advantage at the withinhost level but a fitness disadvantage and low prevalence at the between-host level.

Given the high proportion of mutations in the persistent infections that are deleterious at the between-host level, we were interested in the evolutionary trajectory of mutations at positions in the genome where the baseline consensus in individuals differed from the Wuhan-Hu-1 reference sequence. Of the 236 mutations fulfilling this criteria, 227 reverted to the Wuhan-Hu-1 reference, and 26 of these were recurrent in two or more infections (appendix 1 p 11). Because most of these mutations were also reversions to the major lineage consensus, between-host fitness estimates are not available, but this pattern suggests that at least some of the evolutionary changes towards the Wuhan-Hu-1 reference are likely under strong positive selection.

Of the 379 recurrent mutations, we found 29 highly recurrent mutations in four or more infections (appendix 1 p 11), and seven drug-resistant mutations were found in three or more infections (appendix 1 p 12), and catalogued their known or potential phenotypic properties. The most common mutations were S:N405D (with corresponding nucleotide substitution A22775G in eight infections), NSP14: T516T (T19587A, in 13 infections), and NSP14:C382G (T19183G, in ten infections), all of which were found in persistent omicron infections, BA.2, BA.4, and BA.5. The other frequently recurrent *S* mutations that were found in at least three persistent infections and had very high between-host fitness effects were S:L452R, S:K356T, and S:T547K, all of which are lineage-defining mutations (appendix 1 p 18). In particular, S:K356T is lineage-defining for BA.2.86 and was found in multiple BA.2 and BA.5 persistent infections. On the other hand, most of the recurrent mutations with strong negative between-host fitness effects were concentrated in various non-structural proteins of *ORF1ab* (appendix 1 p 18).

We also investigated potential associations between host characteristics and recurrent mutations in SARS-CoV-2 persistent infections. Specifically, we examined whether there is an association between the age of individuals with persistent infection and the number of times a mutation recurs (appendix 1 p 18), the between-host fitness effect of recurrent mutations and the age group of the individual in which they appeared (appendix 1 p 18), and the fitness effect of the recurrent mutations with respect to the duration of persistent infections (appendix 1 p 18). However, we found no strong associations between these factors.

## Discussion

We characterised viral genomic diversity and within-host evolutionary rates in 576 individuals with persistent SARS-CoV-2 infections, identified through large-scale community surveillance, and including samples collected between November, 2020, and March, 2023. Central to our investigation was the hypothesis that persistent infections with high viral loads could serve as the primary source for the saltatory evolution of the virus at the between-host level, mirroring the same evolutionary changes we see with the emergence of highly divergent variants. This premise led us to identify host characteristics associated with prolonged infections and to characterise viral evolutionary patterns acrossthe genome and betweenindividuals. We were ableto find clear evidence for accelerated virus evolution in the NTD and RBD during some persistent infections, highlighting the importance of identifying and treating such infections to mitigate the risk of emergence of novel highly divergent variants.

We observed significant variability in within-host viral evolutionary rates between infections. This variability was predominantly attributed to the different rates at which individuals accumulated non-synonymous mutations, with the rate of synonymous mutations being similar among all individuals and typically more than four times slower than the rate of non-synonymous mutations. This variability among individuals explains previous findings of limited consensus change mutations in some individuals and overabundance of mutations in others.^10,26^ We also observed considerable variability in non-synonymous evolutionary rates across most of the genome, but not synonymous rates, with the RBD of the spike protein having the highest rate of non-synonymous evolution relative to all other genomic regions. We also observed elevated synonymous rates in the *M* and *N* genes, which might suggest a functional benefit for mRNA stability and translation efficiency, particularly at phosphorylation sites that are abundant in *N*.^11^ However, in the case of *N*, this elevated synonymous divergence can be partially explained by the presence of *ORF9b* as an overlapping reading frame, where 11 synonymous mutations in *N* are non-synonymous with respect to *ORF9b*.

Although older individuals were more likely to experience persistent infections with high viral loads, we found no evidence to suggest that host factors such as age, sex, vaccination status, virus lineage, previous infection, or dynamics of viral RNA titres significantly affected evolutionary rates. Notably, our observation that the within-host evolutionary rates do not significantly differ between vaccinated and unvaccinated individuals suggests that vaccination does not lead to accelerated evolutionary rates. We note, however, that vaccination status does not necessarily equate to the presence of typical vaccine-induced immune responses, since immune responses from previous infections or the persistent infection itself may obscure potential vaccination effects. We did, however, observe a positive association between evolutionary rates and the duration of infection, with longer-lasting infections showing higher rates of non-synonymous evolution. We speculate that individuals with longer infections might have more impaired immune responses, and/or be undergoing treatment, which could result in faster rates of adaptive evolution. While a range of comorbidities, such as diabetes, autoimmune conditions, immune-mediated inflammatory diseases, and other forms of immunosuppression might contribute to persistent SARS-CoV-2 infections, we were not able to investigate this possibility for the persistent infections in our dataset.

Our examination of recurrent within-host mutations which are rare in the general population and have negative between-host fitness effects further illustrates the complex evolutionary dynamics at play within persistent infections. These mutations likely confer a selective advantage within hosts due to enhanced replication rates and/or immune evasion. However, they might prove detrimental at the between-host level, for example if they result in reduced transmissibility of the virus between individuals.^13,27,28^

We found that *ORF6* had the lowest levels of non-synonymous diversity and divergence rate compared with the other genomic regions, indicating it is functionally conserved during persistent infections. Strikingly, we found no diversity in the second codon position of *ORF6*; all mutations at this position would be non-synonymous. These observations are consistent with several studies that have highlighted the crucial role of *ORF6* in viral replication and diseaseprogression.^29^ These resultssuggestthat *ORF6* could be a promising candidate for the development of therapeutic drugs for treating individuals with persistent infections.

A strength of our study is that we were able to detect persistent infections in individuals enrolled in a large community surveillance study, but with this comes limitations since the study was not designed for this purpose. Consequently, we only had a small number of longitudinal samples to assess virus evolutionary dynamics during each infection. Designing a study specifically to identify and follow persistent infections in the community would be very challenging given persistent infections are rare, particularly outside hospital settings, and any specific study would need to be very large to be able to identify sufficient numbers. Another consequence is that sequencing methods were generic, rather than tailored to provide the greater depth ideal to study persistent infections, meaning low-frequency variants might have been missed and duplicate sequencing was not performed as in other studies.^30^ The requirement for sequencing means that we had to focus on persistent infections with high viral loads; plausibly these are most critical for generating diversity. We used information available from the survey on participant characteristics, including vaccination programmes, but, for example, previous infection may have been incompletely ascertained. Finally, given its size and scale, the survey did not collect detailed information on underlying health conditions, so we are not able to investigate these further.

Our findings shed light on the complex interplay between persistent SARS-CoV-2 infections, the demographic characteristics of those infected, and the evolutionary mechanisms driving the virus evolution within these individuals. This study also underscores how persistent infections may contribute to the emergence of highly divergent variants, with factors such asthe duration of infection and accelerated rate of evolution at non-synonymoussites, particularly inthe RBD of the spike protein, influencing their evolutionary rates. Notably, only a small subset of persistent infections showed accelerated rates of viral evolution, and given the absence of strong evidence for onward transmission from persistent infections, the subset of cases that contribute to the generation of new highly divergent variants in the population might be very rare.^16^

## Figures and Tables

**Table:** Baseline characteristics of participants with persistent SARS-CoV-2 infection

	Participants with persistent infection (n=576)
Age, years	65 (45–75)
Sex	
Female	243 (42%)
Male	333 (58%)
Lineage and sampling date	
Alpha	11 (2%); Dec 28, 2020, to Aug 22, 2021
Delta	106 (18%); May 31, 2021, to March 23, 2022
BA.1	102 (18%); Dec 16, 2021, toJuly 26, 2022
BA.2 (excluding BA.2.75)	183 (32%); Dec 29, 2022, to Feb 17, 2023
BA.2.75	21 (4%); Aug 29, 2022, to Feb 26, 2023
BA.4	16 (3%); May 26, 2022, to Dec 3, 2022
BA.5 (excluding BQ.1)	108 (19%); May 30, 2022, to Feb 26, 2023
BQ.1	25 (4%); Sept 16, 2022, to March 1, 2023
XBB	4 (<1%); Nov 10, 2022, to March 6, 2023
Previous vaccination	
Received ≥1 dose	551 (96%)
Not vaccinated	25 (4%)
Previous SARS-CoV-2 infection	
Recorded ≥1 infection	20 (3%)
No previous infection	556 (97%)

Data are median (IQR) for age and n (%) for categorical variables. All percentages shown are relative to the total number of individuals with a persistent infection (n=576).

## Data Availability

This work contains statistical data from the ONS which is Crown Copyright. All raw consensus sequences have been made publicly available as part of the COG-UK Consortium (https://webarchive.nationalarchives.gov.uk/ukgwa/20230505214946/https://www.cogconsortium.uk/priority-areas/data-linkage-analysis/) and are available from the European Nucleotide Archive at EMBL-EBI under accession number PRJEB37886. The accession numbers (COG-IDs) for all individual samples used in our analysis are available in appendix 3.

## Research in context

### Evidence before this study

We reviewed the literature on persistent SARS-CoV-2 infections, focusing on studies published from Jan 1, 2020, to Jan 31, 2024, in PubMed, including search terms “SARS-CoV-2” or “COVID-19” AND “persistent”, “chronic”, “HIV”, “immunocompromised”, “immunodeficient”, or “immunosuppression” in their title, limited only to English-language articles. Our search identified 78 research articles and four review articles documenting SARS-CoV-2 infections in hospitalised patients with conditions such as B-cell dysfunction, autoimmune or autoinflammatory disorders, haematological malignancies, solid organ or haematopoietic stem-cell transplants, or AIDS. These studies primarily focused on characterising clinical outcomes and immune responses in these patients, particularly in relation to antiviral treatments such as monoclonal antibodies, remdesivir, and convalescent plasma. A subset of these studies also examined viral genomic sequences (at the consensus level) collected over the course of SARS-CoV-2 infections, identifying hallmark mutations associated with immune escape or lineage-defining mutations observed in some SARS-CoV-2 variants. However, there is still a gap in the literature for understanding the viral characteristics in individuals with persistent SARS-CoV-2 infection in the general population, particularly those with persistent infections who might not have been hospitalised for COVID-19 or have a documented history of immunosuppression or receiving antiviral treatment for COVID-19. The conditions—both viral and host-related—that facilitate accelerated within-host evolution during persistent infection, potentially leading to the emergence and spread of highly divergent variants, remain largely unexplored.

### Added value of this study

This study provides insights into the within-host evolutionary dynamics of SARS-CoV-2 in individuals with persistent infections with high viral load identified through a large-scale community surveillance study in the UK. This includes both individuals with persistent-chronic infections, which are characterised by consistently high viral loads and positive PCR results throughout the infection, and persistent-rebounding infections, which involve at least one negative PCR result during the course of infection before testing positive again. Unlike previous studies that primarily focused on chronic infections in immunocompromised and hospitalised patients, our research focused on individuals with persistent SARS-CoV-2 infection in the general population. By analysing the genomic samples of SARS-CoV-2 from 576 individuals with persistent infections lasting at least 30 days and characterising the within-host evolutionary dynamics of the virus at the sub-consensus level, we found that these infections were more common in those older than 60 years and in males. We also uncovered significant variability in evolutionary rates among infections, driven predominantly by non-synonymous changes, and found that individuals with longer-lasting infections were more likely to experience accelerated viral evolution. Additionally, we characterised evolutionary forces and fitness effects of mutations both at the within-host and between-host levels, particularly in genomic regions outside the spike gene, which had remained largely unexplored until now. The identification of *ORF6* as a region under strong purifying selection in individuals with persistent SARS-CoV-2 infection offers a new potential therapeutic target.

### Implications of all the available evidence

Persistent SARS-CoV-2 infection in the general population and in hospitalised patients receiving antiviral treatments share important similarities and differences. Both populations show the emergence of hallmark immune escape mutations, with some infections showing accelerated evolution. However, while previous studies have not consistently observed elevated non-synonymous substitution rates in SARS-CoV-2 infections among hospitalised patients, our findings reveal significant variation in evolutionary rates among individuals with persistent infections in the general population, primarily driven by non-synonymous mutations. Moreover, although persistent SARS-CoV-2 infections in both hospitalised patients and those from the general population show a high number of substitutions in the receptor-binding domain of the spike protein and several hallmark mutations linked to treatment resistance, persistent SARS-CoV-2 infections from the general population tend to accumulate a greater number of recurring mutations that are seldom seen in the rest of the population at large. These findings suggest that future surveillance and intervention strategies should account for the distinct evolutionary and transmission dynamics in ongoing infections, both in hospitalised and community settings, to better understand and mitigate the risks associated with variant emergence.
